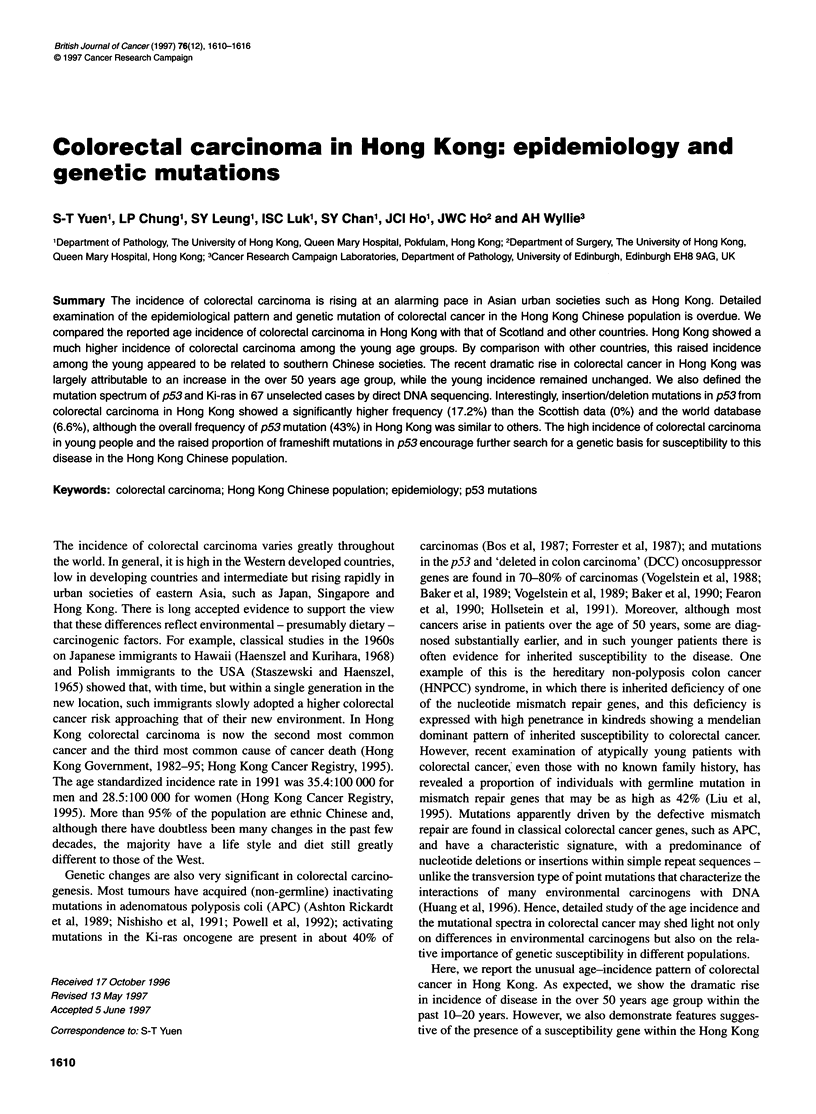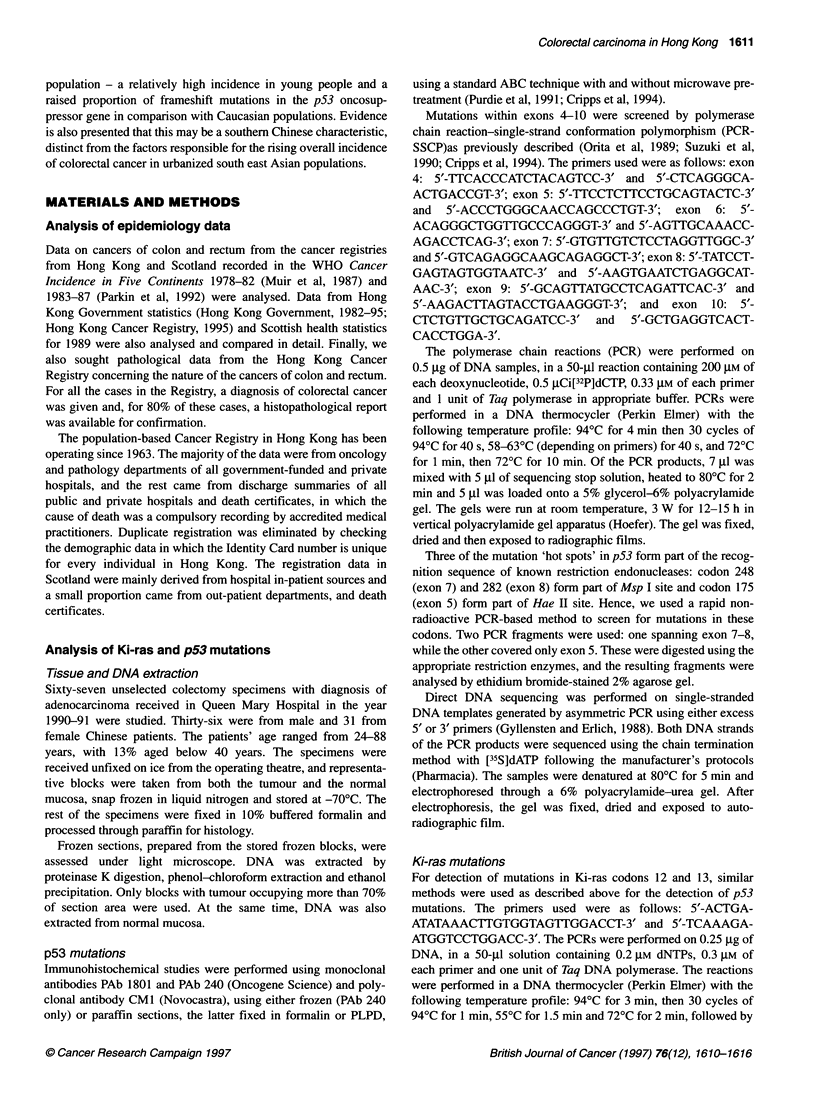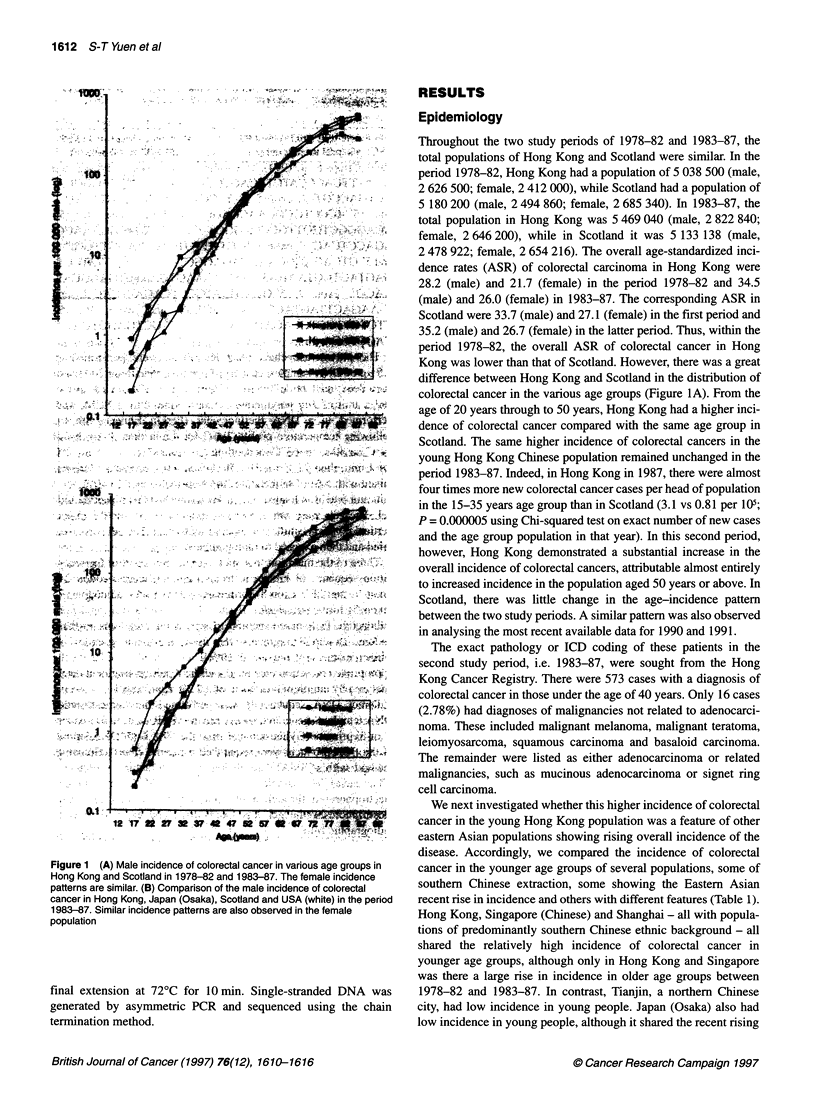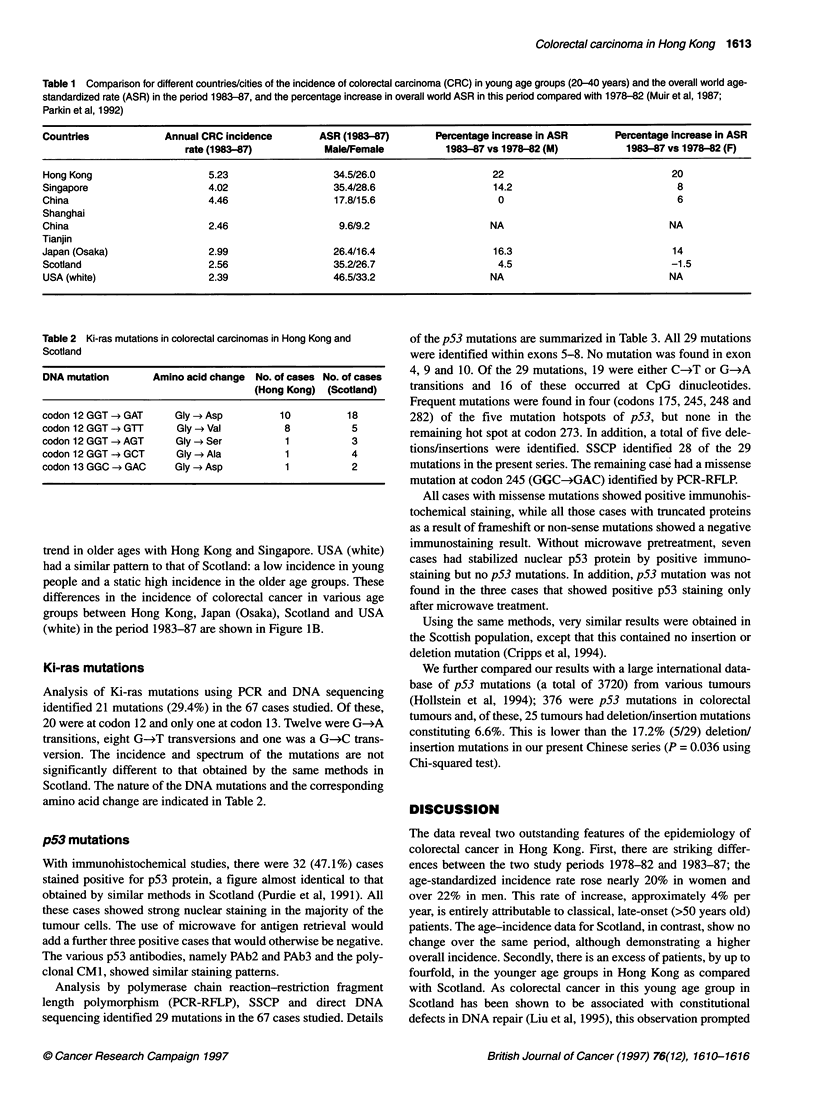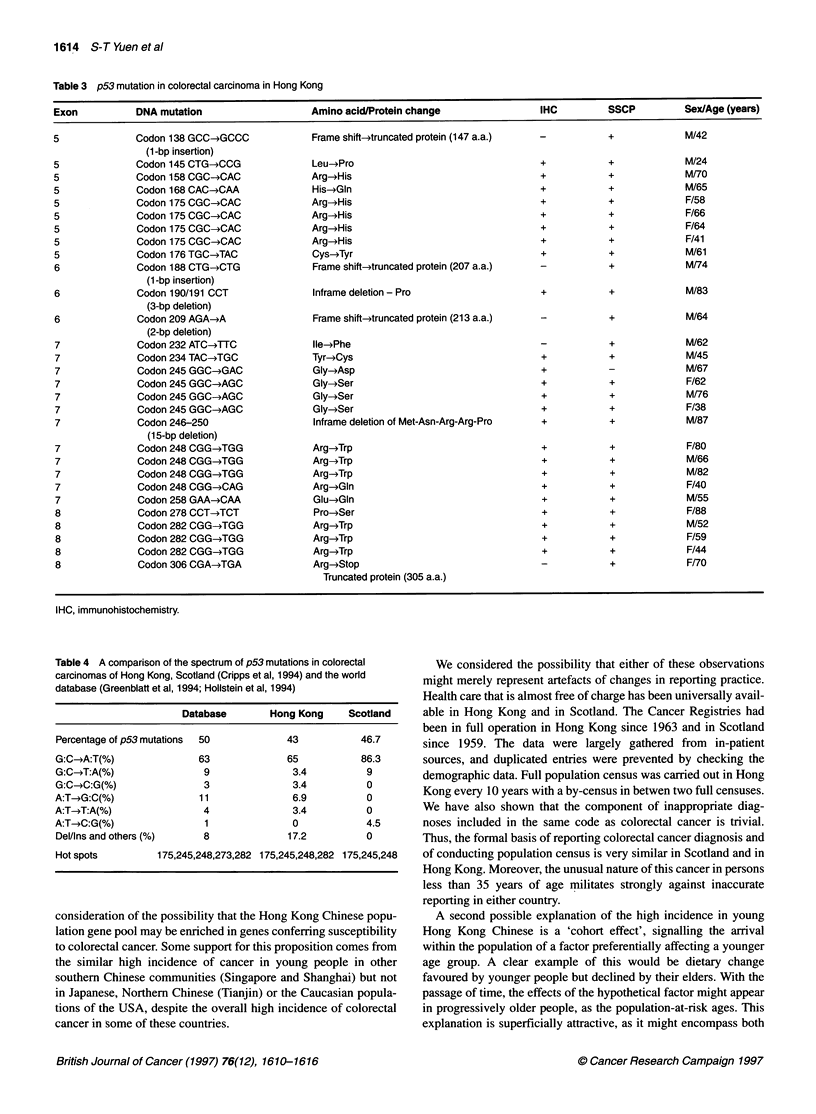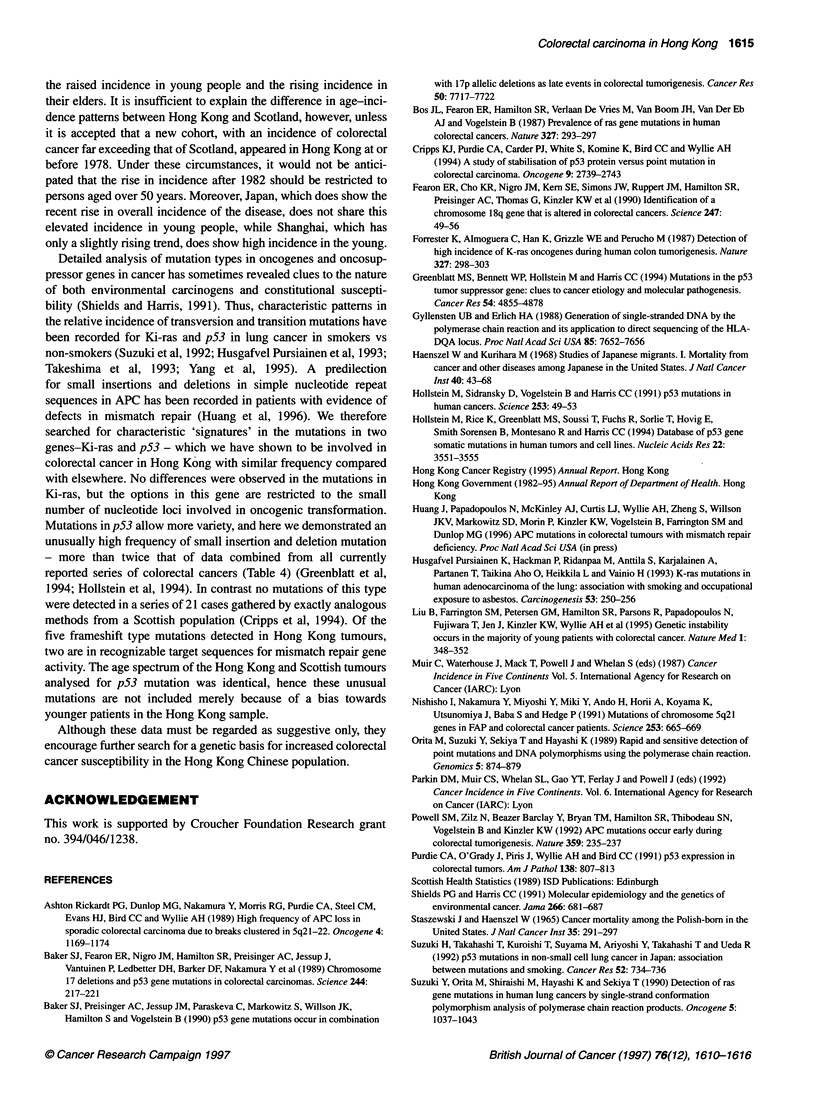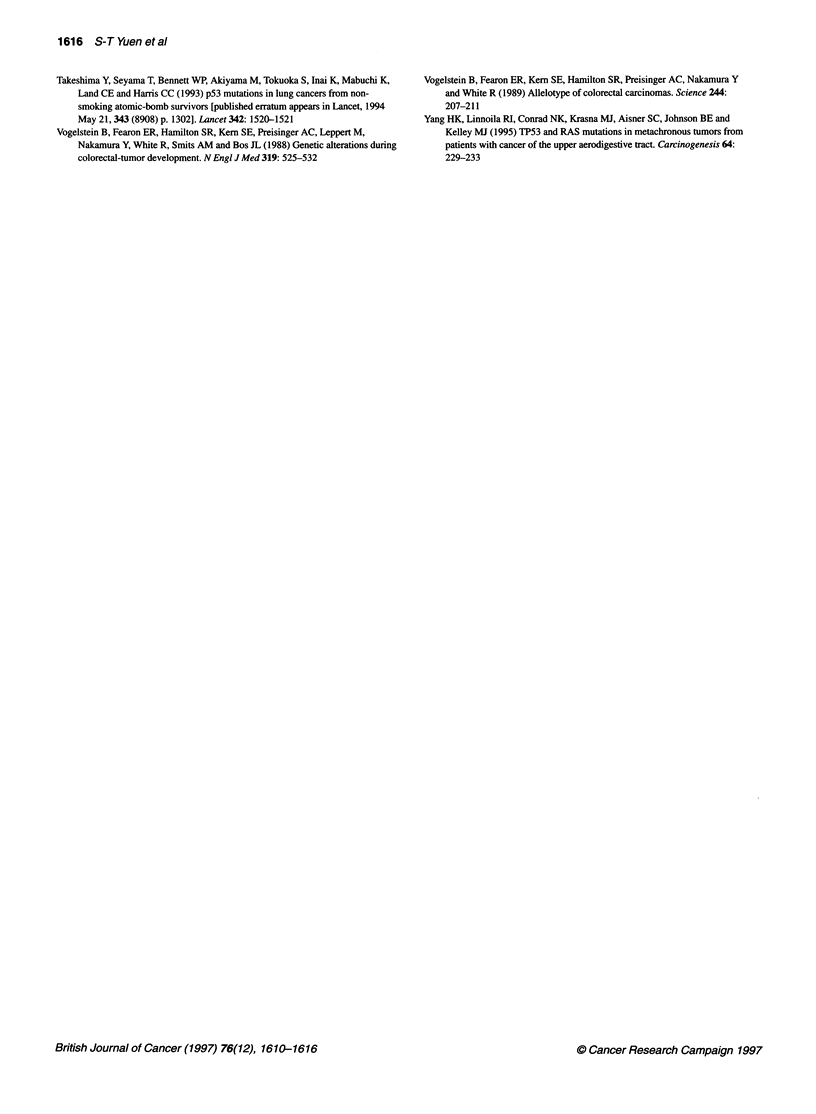# Colorectal carcinoma in Hong Kong: epidemiology and genetic mutations.

**DOI:** 10.1038/bjc.1997.605

**Published:** 1997

**Authors:** S. T. Yuen, L. P. Chung, S. Y. Leung, I. S. Luk, S. Y. Chan, J. C. Ho, J. W. Ho, A. H. Wyllie

**Affiliations:** Department of Pathology, The University of Hong Kong, Queen Mary Hospital, Pokfulam.

## Abstract

The incidence of colorectal carcinoma is rising at an alarming pace in Asian urban societies such as Hong Kong. Detailed examination of the epidemiological pattern and genetic mutation of colorectal cancer in the Hong Kong Chinese population is overdue. We compared the reported age incidence of colorectal carcinoma in Hong Kong with that of Scotland and other countries. Hong Kong showed a much higher incidence of colorectal carcinoma among the young age groups. By comparison with other countries, this raised incidence among the young appeared to be related to southern Chinese societies. The recent dramatic rise in colorectal cancer in Hong Kong was largely attributable to an increase in the over 50 years age group, while the young incidence remained unchanged. We also defined the mutation spectrum of p53 and Ki-ras in 67 unselected cases by direct DNA sequencing. Interestingly, insertion/deletion mutations in p53 from colorectal carcinoma in Hong Kong showed a significantly higher frequency (17.2%) than the Scottish data (0%) and the world database (6.6%), although the overall frequency of p53 mutation (43%) in Hong Kong was similar to others. The high incidence of colorectal carcinoma in young people and the raised proportion of frameshift mutations in p53 encourage further search for a genetic basis for susceptibility to this disease in the Hong Kong Chinese population.


*British Journal of Cancer* (1997) **76**(12), 1610–1616
© 1997 Cancer Research Campaign

### Colorectal carcinoma in Hong Kong: epidemiology and genetic mutations

**S-T Yuen[1], LP Chung[1], SY Leung[1], ISC Luk[1], SY Chan[1], JCI Ho[1], JWC Ho[2] and AH Wyllie[3]**

[1]Department of Pathology, The University of Hong Kong, Queen Mary Hospital, Pokfulam, Hong Kong; [2]Department of Surgery, The University of Hong Kong, Queen Mary Hospital, Hong Kong; [3]Cancer Research Campaign Laboratories, Department of Pathology, University of Edinburgh, Edinburgh EH8 9AG, UK

**Summary** The incidence of colorectal carcinoma is rising at an alarming pace in Asian urban societies such as Hong Kong. Detailed examination of the epidemiological pattern and genetic mutation of colorectal cancer in the Hong Kong Chinese population is overdue. We compared the reported age incidence of colorectal carcinoma in Hong Kong with that of Scotland and other countries. Hong Kong showed a much higher incidence of colorectal carcinoma among the young age groups. By comparison with other countries, this raised incidence among the young appeared to be related to southern Chinese societies. The recent dramatic rise in colorectal cancer in Hong Kong was largely attributable to an increase in the over 50 years age group, while the young incidence remained unchanged. We also defined the mutation spectrum of *p53* and Ki-ras in 67 unselected cases by direct DNA sequencing. Interestingly, insertion/deletion mutations in *p53* from colorectal carcinoma in Hong Kong showed a significantly higher frequency (17.2%) than the Scottish data (0%) and the world database (6.6%), although the overall frequency of *p53* mutation (43%) in Hong Kong was similar to others. The high incidence of colorectal carcinoma in young people and the raised proportion of frameshift mutations in *p53* encourage further search for a genetic basis for susceptibility to this disease in the Hong Kong Chinese population.

**Keywords:** colorectal carcinoma; Hong Kong Chinese population; epidemiology; p53 mutations

The incidence of colorectal carcinoma varies greatly throughout the world. In general, it is high in the Western developed countries, low in developing countries and intermediate but rising rapidly in urban societies of eastern Asia, such as Japan, Singapore and Hong Kong. There is long accepted evidence to support the view that these differences reflect environmental – presumably dietary – carcinogenic factors. For example, classical studies in the 1960s on Japanese immigrants to Hawaii (Haenszel and Kurihara, 1968) and Polish immigrants to the USA (Staszewski and Haenszel, 1965) showed that, with time, but within a single generation in the new location, such immigrants slowly adopted a higher colorectal cancer risk approaching that of their new environment. In Hong Kong colorectal carcinoma is now the second most common cancer and the third most common cause of cancer death (Hong Kong Government, 1982–95; Hong Kong Cancer Registry, 1995). The age standardized incidence rate in 1991 was 35.4:100 000 for men and 28.5:100 000 for women (Hong Kong Cancer Registry, 1995). More than 95% of the population are ethnic Chinese and, although there have doubtless been many changes in the past few decades, the majority have a life style and diet still greatly different to those of the West.

Genetic changes are also very significant in colorectal carcinogenesis. Most tumours have acquired (non-germline) inactivating mutations in adenomatous polyposis coli (APC) (Ashton Rickardt et al, 1989; Nishisho et al, 1991; Powell et al, 1992); activating mutations in the Ki-ras oncogene are present in about 40% of carcinomas (Bos et al, 1987; Forrester et al, 1987); and mutations in the *p53* and 'deleted in colon carcinoma' (DCC) oncosuppressor genes are found in 70–80% of carcinomas (Vogelstein et al, 1988; Baker et al, 1989; Vogelstein et al, 1989; Baker et al, 1990; Fearon et al, 1990; Hollsetein et al, 1991). Moreover, although most cancers arise in patients over the age of 50 years, some are diagnosed substantially earlier, and in such younger patients there is often evidence for inherited susceptibility to the disease. One example of this is the hereditary non-polyposis colon cancer (HNPCC) syndrome, in which there is inherited deficiency of one of the nucleotide mismatch repair genes, and this deficiency is expressed with high penetrance in kindreds showing a mendelian dominant pattern of inherited susceptibility to colorectal cancer. However, recent examination of atypically young patients with colorectal cancer, even those with no known family history, has revealed a proportion of individuals with germline mutation in mismatch repair genes that may be as high as 42% (Liu et al, 1995). Mutations apparently driven by the defective mismatch repair are found in classical colorectal cancer genes, such as APC, and have a characteristic signature, with a predominance of nucleotide deletions or insertions within simple repeat sequences – unlike the transversion type of point mutations that characterize the interactions of many environmental carcinogens with DNA (Huang et al, 1996). Hence, detailed study of the age incidence and the mutational spectra in colorectal cancer may shed light not only on differences in environmental carcinogens but also on the relative importance of genetic susceptibility in different populations.

Here, we report the unusual age–incidence pattern of colorectal cancer in Hong Kong. As expected, we show the dramatic rise in incidence of disease in the over 50 years age group within the past 10–20 years. However, we also demonstrate features suggestive of the presence of a susceptibility gene within the Hong Kong

*Received 17 October 1996*
*Revised 13 May 1997*
*Accepted 5 June 1997*

*Correspondence to:* S-T Yuen population – a relatively high incidence in young people and a raised proportion of frameshift mutations in the *p53* oncosuppressor gene in comparison with Caucasian populations. Evidence is also presented that this may be a southern Chinese characteristic, distinct from the factors responsible for the rising overall incidence of colorectal cancer in urbanized south east Asian populations.

### MATERIALS AND METHODS

### Analysis of epidemiology data

Data on cancers of colon and rectum from the cancer registries from Hong Kong and Scotland recorded in the WHO *Cancer Incidence in Five Continents* 1978–82 (Muir et al, 1987) and 1983–87 (Parkin et al, 1992) were analysed. Data from Hong Kong Government statistics (Hong Kong Government, 1982–95; Hong Kong Cancer Registry, 1995) and Scottish health statistics for 1989 were also analysed and compared in detail. Finally, we also sought pathological data from the Hong Kong Cancer Registry concerning the nature of the cancers of colon and rectum. For all the cases in the Registry, a diagnosis of colorectal cancer was given and, for 80% of these cases, a histopathological report was available for confirmation.

The population-based Cancer Registry in Hong Kong has been operating since 1963. The majority of the data were from oncology and pathology departments of all government-funded and private hospitals, and the rest came from discharge summaries of all public and private hospitals and death certificates, in which the cause of death was a compulsory recording by accredited medical practitioners. Duplicate registration was eliminated by checking the demographic data in which the Identity Card number is unique for every individual in Hong Kong. The registration data in Scotland were mainly derived from hospital in-patient sources and a small proportion came from out-patient departments, and death certificates.

### Analysis of Ki-ras and *p53* mutations

#### *Tissue and DNA extraction*

Sixty-seven unselected colectomy specimens with diagnosis of adenocarcinoma received in Queen Mary Hospital in the year 1990–91 were studied. Thirty-six were from male and 31 from female Chinese patients. The patients' age ranged from 24–88 years, with 13% aged below 40 years. The specimens were received unfixed on ice from the operating theatre, and representative blocks were taken from both the tumour and the normal mucosa, snap frozen in liquid nitrogen and stored at –70°C. The rest of the specimens were fixed in 10% buffered formalin and processed through paraffin for histology.

Frozen sections, prepared from the stored frozen blocks, were assessed under light microscope. DNA was extracted by proteinase K digestion, phenol–chloroform extraction and ethanol precipitation. Only blocks with tumour occupying more than 70% of section area were used. At the same time, DNA was also extracted from normal mucosa.

#### p53 *mutations*

Immunohistochemical studies were performed using monoclonal antibodies PAb 1801 and PAb 240 (Oncogene Science) and polyclonal antibody CM1 (Novocastra), using either frozen (PAb 240 only) or paraffin sections, the latter fixed in formalin or PLPD, using a standard ABC technique with and without microwave pretreatment (Purdie et al, 1991; Cripps et al, 1994).

Mutations within exons 4–10 were screened by polymerase chain reaction–single-strand conformation polymorphism (PCR-SSCP)as previously described (Orita et al, 1989; Suzuki et al, 1990; Cripps et al, 1994). The primers used were as follows: exon 4: 5′-TTCACCCATCTACAGTCC-3′ and 5′-CTCAGGGCA-ACTGACCGT-3′; exon 5: 5′-TTCCTCTTCCTGCAGTACTC-3′ and 5′-ACCCTGGGCAACCAGCCCTGT-3′; exon 6: 5′-ACAGGGCTGGTTGCCCAGGGT-3′ and 5′-AGTTGCAAACC-AGACCTCAG-3′; exon 7: 5′-GTGTTGTCTCCTAGGTTGGC-3′ and 5′-GTCAGAGGCAAGCAGAGGCT-3′; exon 8: 5′-TATCCT-GAGTAGTGGTAATC-3′ and 5′-AAGTGAATCTGAGGCAT-AAC-3′; exon 9: 5′-GCAGTTATGCCTCAGATTCAC-3′ and 5′-AAGACTTAGTACCTGAAGGGT-3′; and exon 10: 5′-CTCTGTTGCTGCAGATCC-3′ and 5′-GCTGAGGTCACT-CACCTGGA-3′.

The polymerase chain reactions (PCR) were performed on 0.5 μg of DNA samples, in a 50-μl reaction containing 200 μM of each deoxynucleotide, 0.5 μCi[$^{32}$P]dCTP, 0.33 μM of each primer and 1 unit of *Taq* polymerase in appropriate buffer. PCRs were performed in a DNA thermocycler (Perkin Elmer) with the following temperature profile: 94°C for 4 min then 30 cycles of 94°C for 40 s, 58–63°C (depending on primers) for 40 s, and 72°C for 1 min, then 72°C for 10 min. Of the PCR products, 7 μl was mixed with 5 μl of sequencing stop solution, heated to 80°C for 2 min and 5 μl was loaded onto a 5% glycerol–6% polyacrylamide gel. The gels were run at room temperature, 3 W for 12–15 h in vertical polyacrylamide gel apparatus (Hoefer). The gel was fixed, dried and then exposed to radiographic films.

Three of the mutation 'hot spots' in *p53* form part of the recognition sequence of known restriction endonucleases: codon 248 (exon 7) and 282 (exon 8) form part of *Msp* I site and codon 175 (exon 5) form part of *Hae* II site. Hence, we used a rapid non-radioactive PCR-based method to screen for mutations in these codons. Two PCR fragments were used: one spanning exon 7–8, while the other covered only exon 5. These were digested using the appropriate restriction enzymes, and the resulting fragments were analysed by ethidium bromide-stained 2% agarose gel.

Direct DNA sequencing was performed on single-stranded DNA templates generated by asymmetric PCR using either excess 5′ or 3′ primers (Gyllensten and Erlich, 1988). Both DNA strands of the PCR products were sequenced using the chain termination method with [$^{35}$S]dATP following the manufacturer's protocols (Pharmacia). The samples were denatured at 80°C for 5 min and electrophoresed through a 6% polyacrylamide–urea gel. After electrophoresis, the gel was fixed, dried and exposed to autoradiographic film.

#### *Ki-ras mutations*

For detection of mutations in Ki-ras codons 12 and 13, similar methods were used as described above for the detection of *p53* mutations. The primers used were as follows: 5′-ACTGA-ATATAAACTTGTGGTAGTTGGACCT-3′ and 5′-TCAAAGA-ATGGTCCTGGACC-3′. The PCRs were performed on 0.25 μg of DNA, in a 50-μl solution containing 0.2 μM dNTPs, 0.3 μM of each primer and one unit of *Taq* DNA polymerase. The reactions were performed in a DNA thermocycler (Perkin Elmer) with the following temperature profile: 94°C for 3 min, then 30 cycles of 94°C for 1 min, 55°C for 1.5 min and 72°C for 2 min, followed by

© Cancer Research Campaign 1997

**Figure 1** (**A**) Male incidence of colorectal cancer in various age groups in Hong Kong and Scotland in 1978–82 and 1983–87. The female incidence patterns are similar. (**B**) Comparison of the male incidence of colorectal cancer in Hong Kong, Japan (Osaka), Scotland and USA (white) in the period 1983–87. Similar incidence patterns are also observed in the female population final extension at 72°C for 10 min. Single-stranded DNA was generated by asymmetric PCR and sequenced using the chain termination method.

### RESULTS

### Epidemiology

Throughout the two study periods of 1978–82 and 1983–87, the total populations of Hong Kong and Scotland were similar. In the period 1978–82, Hong Kong had a population of 5 038 500 (male, 2 626 500; female, 2 412 000), while Scotland had a population of 5 180 200 (male, 2 494 860; female, 2 685 340). In 1983–87, the total population in Hong Kong was 5 469 040 (male, 2 822 840; female, 2 646 200), while in Scotland it was 5 133 138 (male, 2 478 922; female, 2 654 216). The overall age-standardized incidence rates (ASR) of colorectal carcinoma in Hong Kong were 28.2 (male) and 21.7 (female) in the period 1978–82 and 34.5 (male) and 26.0 (female) in 1983–87. The corresponding ASR in Scotland were 33.7 (male) and 27.1 (female) in the first period and 35.2 (male) and 26.7 (female) in the latter period. Thus, within the period 1978–82, the overall ASR of colorectal cancer in Hong Kong was lower than that of Scotland. However, there was a great difference between Hong Kong and Scotland in the distribution of colorectal cancer in the various age groups (Figure 1A). From the age of 20 years through to 50 years, Hong Kong had a higher incidence of colorectal cancer compared with the same age group in Scotland. The same higher incidence of colorectal cancers in the young Hong Kong Chinese population remained unchanged in the period 1983–87. Indeed, in Hong Kong in 1987, there were almost four times more new colorectal cancer cases per head of population in the 15–35 years age group than in Scotland (3.1 vs 0.81 per $10^5$; $P = 0.000005$ using Chi-squared test on exact number of new cases and the age group population in that year). In this second period, however, Hong Kong demonstrated a substantial increase in the overall incidence of colorectal cancers, attributable almost entirely to increased incidence in the population aged 50 years or above. In Scotland, there was little change in the age–incidence pattern between the two study periods. A similar pattern was also observed in analysing the most recent available data for 1990 and 1991.

The exact pathology or ICD coding of these patients in the second study period, i.e. 1983–87, were sought from the Hong Kong Cancer Registry. There were 573 cases with a diagnosis of colorectal cancer in those under the age of 40 years. Only 16 cases (2.78%) had diagnoses of malignancies not related to adenocarcinoma. These included malignant melanoma, malignant teratoma, leiomyosarcoma, squamous carcinoma and basaloid carcinoma. The remainder were listed as either adenocarcinoma or related malignancies, such as mucinous adenocarcinoma or signet ring cell carcinoma.

We next investigated whether this higher incidence of colorectal cancer in the young Hong Kong population was a feature of other eastern Asian populations showing rising overall incidence of the disease. Accordingly, we compared the incidence of colorectal cancer in the younger age groups of several populations, some of southern Chinese extraction, some showing the Eastern Asian recent rise in incidence and others with different features (Table 1). Hong Kong, Singapore (Chinese) and Shanghai – all with populations of predominantly southern Chinese ethnic background – all shared the relatively high incidence of colorectal cancer in younger age groups, although only in Hong Kong and Singapore was there a large rise in incidence in older age groups between 1978–82 and 1983–87. In contrast, Tianjin, a northern Chinese city, had low incidence in young people. Japan (Osaka) also had low incidence in young people, although it shared the recent rising

© Cancer Research Campaign 1997

**Table 1** Comparison for different countries/cities of the incidence of colorectal carcinoma (CRC) in young age groups (20–40 years) and the overall world age-standardized rate (ASR) in the period 1983–87, and the percentage increase in overall world ASR in this period compared with 1978–82 (Muir et al, 1987; Parkin et al, 1992)

| Countries | Annual CRC incidence rate (1983–87) | ASR (1983–87) Male/Female | Percentage increase in ASR 1983–87 vs 1978–82 (M) | Percentage increase in ASR 1983–87 vs 1978–82 (F) |
|---|---|---|---|---|
| Hong Kong | 5.23 | 34.5/26.0 | 22 | 20 |
| Singapore | 4.02 | 35.4/28.6 | 14.2 | 8 |
| China Shanghai | 4.46 | 17.8/15.6 | 0 | 6 |
| China Tianjin | 2.46 | 9.6/9.2 | NA | NA |
| Japan (Osaka) | 2.99 | 26.4/16.4 | 16.3 | 14 |
| Scotland | 2.56 | 35.2/26.7 | 4.5 | –1.5 |
| USA (white) | 2.39 | 46.5/33.2 | NA | NA |

**Table 2** Ki-ras mutations in colorectal carcinomas in Hong Kong and Scotland

| DNA mutation | Amino acid change | No. of cases (Hong Kong) | No. of cases (Scotland) |
|---|---|---|---|
| codon 12 GGT → GAT | Gly → Asp | 10 | 18 |
| codon 12 GGT → GTT | Gly → Val | 8 | 5 |
| codon 12 GGT → AGT | Gly → Ser | 1 | 3 |
| codon 12 GGT → GCT | Gly → Ala | 1 | 4 |
| codon 13 GGC → GAC | Gly → Asp | 1 | 2 |

trend in older ages with Hong Kong and Singapore. USA (white) had a similar pattern to that of Scotland: a low incidence in young people and a static high incidence in the older age groups. These differences in the incidence of colorectal cancer in various age groups between Hong Kong, Japan (Osaka), Scotland and USA (white) in the period 1983–87 are shown in Figure 1B.

### Ki-ras mutations

Analysis of Ki-ras mutations using PCR and DNA sequencing identified 21 mutations (29.4%) in the 67 cases studied. Of these, 20 were at codon 12 and only one at codon 13. Twelve were G→A transitions, eight G→T transversions and one was a G→C transversion. The incidence and spectrum of the mutations are not significantly different to that obtained by the same methods in Scotland. The nature of the DNA mutations and the corresponding amino acid change are indicated in Table 2.

### *p53* mutations

With immunohistochemical studies, there were 32 (47.1%) cases stained positive for p53 protein, a figure almost identical to that obtained by similar methods in Scotland (Purdie et al, 1991). All these cases showed strong nuclear staining in the majority of the tumour cells. The use of microwave for antigen retrieval would add a further three positive cases that would otherwise be negative. The various p53 antibodies, namely PAb2 and PAb3 and the polyclonal CM1, showed similar staining patterns.

Analysis by polymerase chain reaction–restriction fragment length polymorphism (PCR-RFLP), SSCP and direct DNA sequencing identified 29 mutations in the 67 cases studied. Details of the *p53* mutations are summarized in Table 3. All 29 mutations were identified within exons 5–8. No mutation was found in exon 4, 9 and 10. Of the 29 mutations, 19 were either C→T or G→A transitions and 16 of these occurred at CpG dinucleotides. Frequent mutations were found in four (codons 175, 245, 248 and 282) of the five mutation hotspots of *p53*, but none in the remaining hot spot at codon 273. In addition, a total of five deletions/insertions were identified. SSCP identified 28 of the 29 mutations in the present series. The remaining case had a missense mutation at codon 245 (GGC→GAC) identified by PCR-RFLP.

All cases with missense mutations showed positive immunohistochemical staining, while all those cases with truncated proteins as a result of frameshift or non-sense mutations showed a negative immunostaining result. Without microwave pretreatment, seven cases had stabilized nuclear p53 protein by positive immunostaining but no *p53* mutations. In addition, *p53* mutation was not found in the three cases that showed positive p53 staining only after microwave treatment.

Using the same methods, very similar results were obtained in the Scottish population, except that this contained no insertion or deletion mutation (Cripps et al, 1994).

We further compared our results with a large international database of *p53* mutations (a total of 3720) from various tumours (Hollstein et al, 1994); 376 were *p53* mutations in colorectal tumours and, of these, 25 tumours had deletion/insertion mutations constituting 6.6%. This is lower than the 17.2% (5/29) deletion/insertion mutations in our present Chinese series ($P = 0.036$ using Chi-squared test).

### DISCUSSION

The data reveal two outstanding features of the epidemiology of colorectal cancer in Hong Kong. First, there are striking differences between the two study periods 1978–82 and 1983–87; the age-standardized incidence rate rose nearly 20% in women and over 22% in men. This rate of increase, approximately 4% per year, is entirely attributable to classical, late-onset (>50 years old) patients. The age–incidence data for Scotland, in contrast, show no change over the same period, although demonstrating a higher overall incidence. Secondly, there is an excess of patients, by up to fourfold, in the younger age groups in Hong Kong as compared with Scotland. As colorectal cancer in this young age group in Scotland has been shown to be associated with constitutional defects in DNA repair (Liu et al, 1995), this observation prompted

© Cancer Research Campaign 1997 

**Table 3** *p53* mutation in colorectal carcinoma in Hong Kong

| Exon | DNA mutation | Amino acid/Protein change | IHC | SSCP | Sex/Age (years) |
|---|---|---|---|---|---|
| 5 | Codon 138 GCC→GCCC (1-bp insertion) | Frame shift→truncated protein (147 a.a.) | – | + | M/42 |
| 5 | Codon 145 CTG→CCG | Leu→Pro | + | + | M/24 |
| 5 | Codon 158 CGC→CAC | Arg→His | + | + | M/70 |
| 5 | Codon 168 CAC→CAA | His→Gln | + | + | M/65 |
| 5 | Codon 175 CGC→CAC | Arg→His | + | + | F/58 |
| 5 | Codon 175 CGC→CAC | Arg→His | + | + | F/66 |
| 5 | Codon 175 CGC→CAC | Arg→His | + | + | F/64 |
| 5 | Codon 175 CGC→CAC | Arg→His | + | + | F/41 |
| 5 | Codon 176 TGC→TAC | Cys→Tyr | + | + | M/61 |
| 6 | Codon 188 CTG→CTG (1-bp insertion) | Frame shift→truncated protein (207 a.a.) | – | + | M/74 |
| 6 | Codon 190/191 CCT (3-bp deletion) | Inframe deletion – Pro | + | + | M/83 |
| 6 | Codon 209 AGA→A (2-bp deletion) | Frame shift→truncated protein (213 a.a.) | – | + | M/64 |
| 7 | Codon 232 ATC→TTC | Ile→Phe | – | + | M/62 |
| 7 | Codon 234 TAC→TGC | Tyr→Cys | + | + | M/45 |
| 7 | Codon 245 GGC→GAC | Gly→Asp | + | – | M/67 |
| 7 | Codon 245 GGC→AGC | Gly→Ser | + | + | F/62 |
| 7 | Codon 245 GGC→AGC | Gly→Ser | + | + | M/76 |
| 7 | Codon 245 GGC→AGC | Gly→Ser | + | + | F/38 |
| 7 | Codon 246–250 (15-bp deletion) | Inframe deletion of Met-Asn-Arg-Arg-Pro | + | + | M/87 |
| 7 | Codon 248 CGG→TGG | Arg→Trp | + | + | F/80 |
| 7 | Codon 248 CGG→TGG | Arg→Trp | + | + | M/66 |
| 7 | Codon 248 CGG→TGG | Arg→Trp | + | + | M/82 |
| 7 | Codon 248 CGG→CAG | Arg→Gln | + | + | F/40 |
| 7 | Codon 258 GAA→CAA | Glu→Gln | + | + | M/55 |
| 8 | Codon 278 CCT→TCT | Pro→Ser | + | + | F/88 |
| 8 | Codon 282 CGG→TGG | Arg→Trp | + | + | M/52 |
| 8 | Codon 282 CGG→TGG | Arg→Trp | + | + | F/59 |
| 8 | Codon 282 CGG→TGG | Arg→Trp | + | + | F/44 |
| 8 | Codon 306 CGA→TGA | Arg→Stop Truncated protein (305 a.a.) | – | + | F/70 |

IHC, immunohistochemistry.

**Table 4** A comparison of the spectrum of *p53* mutations in colorectal carcinomas of Hong Kong, Scotland (Cripps et al, 1994) and the world database (Greenblatt et al, 1994; Hollstein et al, 1994)

| | Database | Hong Kong | Scotland |
|---|---|---|---|
| Percentage of *p53* mutations | 50 | 43 | 46.7 |
| G:C→A:T(%) | 63 | 65 | 86.3 |
| G:C→T:A(%) | 9 | 3.4 | 9 |
| G:C→C:G(%) | 3 | 3.4 | 0 |
| A:T→G:C(%) | 11 | 6.9 | 0 |
| A:T→T:A(%) | 4 | 3.4 | 0 |
| A:T→C:G(%) | 1 | 0 | 4.5 |
| Del/Ins and others (%) | 8 | 17.2 | 0 |
| Hot spots | 175,245,248,273,282 | 175,245,248,282 | 175,245,248 |

consideration of the possibility that the Hong Kong Chinese population gene pool may be enriched in genes conferring susceptibility to colorectal cancer. Some support for this proposition comes from the similar high incidence of cancer in young people in other southern Chinese communities (Singapore and Shanghai) but not in Japanese, Northern Chinese (Tianjin) or the Caucasian populations of the USA, despite the overall high incidence of colorectal cancer in some of these countries.

We considered the possibility that either of these observations might merely represent artefacts of changes in reporting practice. Health care that is almost free of charge has been universally available in Hong Kong and in Scotland. The Cancer Registries had been in full operation in Hong Kong since 1963 and in Scotland since 1959. The data were largely gathered from in-patient sources, and duplicated entries were prevented by checking the demographic data. Full population census was carried out in Hong Kong every 10 years with a by-census in betwen two full censuses. We have also shown that the component of inappropriate diagnoses included in the same code as colorectal cancer is trivial. Thus, the formal basis of reporting colorectal cancer diagnosis and of conducting population census is very similar in Scotland and in Hong Kong. Moreover, the unusual nature of this cancer in persons less than 35 years of age militates strongly against inaccurate reporting in either country.

A second possible explanation of the high incidence in young Hong Kong Chinese is a 'cohort effect', signalling the arrival within the population of a factor preferentially affecting a younger age group. A clear example of this would be dietary change favoured by younger people but declined by their elders. With the passage of time, the effects of the hypothetical factor might appear in progressively older people, as the population-at-risk ages. This explanation is superficially attractive, as it might encompass both

 © Cancer Research Campaign 1997

the raised incidence in young people and the rising incidence in their elders. It is insufficient to explain the difference in age–incidence patterns between Hong Kong and Scotland, however, unless it is accepted that a new cohort, with an incidence of colorectal cancer far exceeding that of Scotland, appeared in Hong Kong at or before 1978. Under these circumstances, it would not be anticipated that the rise in incidence after 1982 should be restricted to persons aged over 50 years. Moreover, Japan, which does show the recent rise in overall incidence of the disease, does not share this elevated incidence in young people, while Shanghai, which has only a slightly rising trend, does show high incidence in the young.

Detailed analysis of mutation types in oncogenes and oncosuppressor genes in cancer has sometimes revealed clues to the nature of both environmental carcinogens and constitutional susceptibility (Shields and Harris, 1991). Thus, characteristic patterns in the relative incidence of transversion and transition mutations have been recorded for Ki-ras and *p53* in lung cancer in smokers vs non-smokers (Suzuki et al, 1992; Husgafvel Pursiainen et al, 1993; Takeshima et al, 1993; Yang et al, 1995). A predilection for small insertions and deletions in simple nucleotide repeat sequences in APC has been recorded in patients with evidence of defects in mismatch repair (Huang et al, 1996). We therefore searched for characteristic 'signatures' in the mutations in two genes–Ki-ras and *p53* – which we have shown to be involved in colorectal cancer in Hong Kong with similar frequency compared with elsewhere. No differences were observed in the mutations in Ki-ras, but the options in this gene are restricted to the small number of nucleotide loci involved in oncogenic transformation. Mutations in *p53* allow more variety, and here we demonstrated an unusually high frequency of small insertion and deletion mutation – more than twice that of data combined from all currently reported series of colorectal cancers (Table 4) (Greenblatt et al, 1994; Hollstein et al, 1994). In contrast no mutations of this type were detected in a series of 21 cases gathered by exactly analogous methods from a Scottish population (Cripps et al, 1994). Of the five frameshift type mutations detected in Hong Kong tumours, two are in recognizable target sequences for mismatch repair gene activity. The age spectrum of the Hong Kong and Scottish tumours analysed for *p53* mutation was identical, hence these unusual mutations are not included merely because of a bias towards younger patients in the Hong Kong sample.

Although these data must be regarded as suggestive only, they encourage further search for a genetic basis for increased colorectal cancer susceptibility in the Hong Kong Chinese population.

### ACKNOWLEDGEMENT

This work is supported by Croucher Foundation Research grant no. 394/046/1238.

### REFERENCES

Ashton Rickardt PG, Dunlop MG, Nakamura Y, Morris RG, Purdie CA, Steel CM, Evans HJ, Bird CC and Wyllie AH (1989) High frequency of APC loss in sporadic colorectal carcinoma due to breaks clustered in 5q21–22. *Oncogene* **4**: 1169–1174

Baker SJ, Fearon ER, Nigro JM, Hamilton SR, Preisinger AC, Jessup J, Vantuinen P, Ledbetter DH, Barker DF, Nakamura Y et al (1989) Chromosome 17 deletions and p53 gene mutations in colorectal carcinomas. *Science* **244**: 217–221

Baker SJ, Preisinger AC, Jessup JM, Paraskeva C, Markowitz S, Willson JK, Hamilton S and Vogelstein B (1990) p53 gene mutations occur in combination with 17p allelic deletions as late events in colorectal tumorigenesis. *Cancer Res* **50**: 7717–7722

Bos JL, Fearon ER, Hamilton SR, Verlaan De Vries M, Van Boom JH, Van Der Eb AJ and Vogelstein B (1987) Prevalence of ras gene mutations in human colorectal cancers. *Nature* **327**: 293–297

Cripps KJ, Purdie CA, Carder PJ, White S, Komine K, Bird CC and Wyllie AH (1994) A study of stabilisation of p53 protein versus point mutation in colorectal carcinoma. *Oncogene* **9**: 2739–2743

Fearon ER, Cho KR, Nigro JM, Kern SE, Simons JW, Ruppert JM, Hamilton SR, Preisinger AC, Thomas G, Kinzler KW et al (1990) Identification of a chromosome 18q gene that is altered in colorectal cancers. *Science* **247**: 49–56

Forrester K, Almoguera C, Han K, Grizzle WE and Perucho M (1987) Detection of high incidence of K-ras oncogenes during human colon tumorigenesis. *Nature* **327**: 298–303

Greenblatt MS, Bennett WP, Hollstein M and Harris CC (1994) Mutations in the p53 tumor suppressor gene: clues to cancer etiology and molecular pathogenesis. *Cancer Res* **54**: 4855–4878

Gyllensten UB and Erlich HA (1988) Generation of single-stranded DNA by the polymerase chain reaction and its application to direct sequencing of the HLA-DQA locus. *Proc Natl Acad Sci USA* **85**: 7652–7656

Haenszel W and Kurihara M (1968) Studies of Japanese migrants. I. Mortality from cancer and other diseases among Japanese in the United States. *J Natl Cancer Inst* **40**: 43–68

Hollstein M, Sidransky D, Vogelstein B and Harris CC (1991) p53 mutations in human cancers. *Science* **253**: 49–53

Hollstein M, Rice K, Greenblatt MS, Soussi T, Fuchs R, Sorlie T, Hovig E, Smith Sorensen B, Montesano R and Harris CC (1994) Database of p53 gene somatic mutations in human tumors and cell lines. *Nucleic Acids Res* **22**: 3551–3555

Hong Kong Cancer Registry (1995) *Annual Report*. Hong Kong

Hong Kong Government (1982–95) *Annual Report of Department of Health*. Hong Kong

Huang J, Papadopoulos N, McKinley AJ, Curtis LJ, Wyllie AH, Zheng S, Willson JKV, Markowitz SD, Morin P, Kinzler KW, Vogelstein B, Farrington SM and Dunlop MG (1996) APC mutations in colorectal tumours with mismatch repair deficiency. *Proc Natl Acad Sci USA* (in press)

Husgafvel Pursiainen K, Hackman P, Ridanpaa M, Anttila S, Karjalainen A, Partanen T, Taikina Aho O, Heikkila L and Vainio H (1993) K-ras mutations in human adenocarcinoma of the lung: association with smoking and occupational exposure to asbestos. *Carcinogenesis* **53**: 250–256

Liu B, Farrington SM, Petersen GM, Hamilton SR, Parsons R, Papadopoulos N, Fujiwara T, Jen J, Kinzler KW, Wyllie AH et al (1995) Genetic instability occurs in the majority of young patients with colorectal cancer. *Nature Med* **1**: 348–352

Muir C, Waterhouse J, Mack T, Powell J and Whelan S (eds) (1987) *Cancer Incidence in Five Continents* Vol. 5. International Agency for Research on Cancer (IARC): Lyon

Nishisho I, Nakamura Y, Miyoshi Y, Miki Y, Ando H, Horii A, Koyama K, Utsunomiya J, Baba S and Hedge P (1991) Mutations of chromosome 5q21 genes in FAP and colorectal cancer patients. *Science* **253**: 665–669

Orita M, Suzuki Y, Sekiya T and Hayashi K (1989) Rapid and sensitive detection of point mutations and DNA polymorphisms using the polymerase chain reaction. *Genomics* **5**: 874–879

Parkin DM, Muir CS, Whelan SL, Gao YT, Ferlay J and Powell J (eds) (1992) *Cancer Incidence in Five Continents*. Vol. 6. International Agency for Research on Cancer (IARC): Lyon

Powell SM, Zilz N, Beazer Barclay Y, Bryan TM, Hamilton SR, Thibodeau SN, Vogelstein B and Kinzler KW (1992) APC mutations occur early during colorectal tumorigenesis. *Nature* **359**: 235–237

Purdie CA, O'Grady J, Piris J, Wyllie AH and Bird CC (1991) p53 expression in colorectal tumors. *Am J Pathol* **138**: 807–813

Scottish Health Statistics (1989) ISD Publications: Edinburgh

Shields PG and Harris CC (1991) Molecular epidemiology and the genetics of environmental cancer. *Jama* **266**: 681–687

Staszewski J and Haenszel W (1965) Cancer mortality among the Polish-born in the United States. *J Natl Cancer Inst* **35**: 291–297

Suzuki H, Takahashi T, Kuroishi T, Suyama M, Ariyoshi Y, Takahashi T and Ueda R (1992) p53 mutations in non-small cell lung cancer in Japan: association between mutations and smoking. *Cancer Res* **52**: 734–736

Suzuki Y, Orita M, Shiraishi M, Hayashi K and Sekiya T (1990) Detection of ras gene mutations in human lung cancers by single-strand conformation polymorphism analysis of polymerase chain reaction products. *Oncogene* **5**: 1037–1043

© Cancer Research Campaign 1997

Takeshima Y, Seyama T, Bennett WP, Akiyama M, Tokuoka S, Inai K, Mabuchi K, Land CE and Harris CC (1993) p53 mutations in lung cancers from non-smoking atomic-bomb survivors [published erratum appears in Lancet, 1994 May 21, **343** (8908) p. 1302]. *Lancet* **342**: 1520–1521

Vogelstein B, Fearon ER, Hamilton SR, Kern SE, Preisinger AC, Leppert M, Nakamura Y, White R, Smits AM and Bos JL (1988) Genetic alterations during colorectal-tumor development. *N Engl J Med* **319**: 525–532

Vogelstein B, Fearon ER, Kern SE, Hamilton SR, Preisinger AC, Nakamura Y and White R (1989) Allelotype of colorectal carcinomas. *Science* **244**: 207–211

Yang HK, Linnoila RI, Conrad NK, Krasna MJ, Aisner SC, Johnson BE and Kelley MJ (1995) TP53 and RAS mutations in metachronous tumors from patients with cancer of the upper aerodigestive tract. *Carcinogenesis* **64**: 229–233

© Cancer Research Campaign 1997